# “Dismantling Fixed Time, Variable Outcome Education: Abandoning ‘Ready or Not, Here they Come’ is Overdue”

**DOI:** 10.5334/pme.10

**Published:** 2023-03-13

**Authors:** Carol Carraccio, Alison Lentz, Daniel J. Schumacher

**Affiliations:** 1 Google Research, US; 2 Cincinnati Children’s Hospital Medical Center and University of Cincinnati College of Medicine, Cincinnati, Ohio, US

## Abstract

Two decades after competency-based medical education appeared in the lexicon of medical educators, the community continues to struggle with realizing its full potential. The implementation of the time variable, fixed outcome component has languished based on complexity compounded by resistance to change. Learners continue to transition from medical school to residency, and then practice, primarily based on time rather than having achieved the ability to meet the needs of the patient populations they will serve. Only those few who demonstrate glaring deficiencies do not graduate.

The authors urge the medical education community to move from the current fixed time path of medical education toward the implementation of a true continuum of time variable, fixed outcome education, training, and *deliberate* practice. The latter is defined by purposeful learning, coaching, feedback, and repetition on the path to achieving and maintaining expertise. The opportunities afforded by such a time-variable, fixed outcome approach include: 1) development of a career long growth mindset, 2) ability to address evolving population health needs and careers within the context of one’s practice, and 3) continual improvement of care quality and outcomes for patients on the journey towards expertise for providers.

*“Our educational system….is a somewhat mad world in which we hold time as a constant and allow achievement to be variable.”* [1]

Two decades have elapsed since the introduction of competency-based medical education [2,3]. Although bits and pieces of this educational paradigm have been implemented across countries and systems, the key principles of time variability and fixed outcomes have languished. Resistance to change combined with the complexity of the paradigm shift are likely major culprits. At the completion of training stages, medical schools and clinical competency committees in postgraduate training make high stakes summative decisions as to whether learners are “ready or not” to graduate [4,5]. These decisions should be based on predetermined learner outcomes and grounded in both observed competence and perceived future capacity to provide safe, effective care in unfamiliar situations [6]. However, these decisions often default to allowing transitions, based mainly on insufficient evidence to challenge them [6]. Typically, opposing forces only arise from gross clinical incompetence or egregious unprofessional behavior given the inability to support and finance meaningful remediation efforts [7]. Despite misgivings, time remains the major determinant of transition. As detailed in this paper, we urge the medical education community to consider the benefits of moving from the current fixed time path of becoming a physician toward a true continuum of time variable, fixed outcome education, training, and *deliberate* practice [8,9].

## The status quo: Reflections on making ready or not decisions in our current fixed time, variable outcome approach

Currently, ready or not summative decisions are made at predetermined time intervals during education and training. This is antithetical to the well-established truth that learning is a socially enabled developmental process with fits and spurts and reasonable ranges of time during which most people actually reach the state of “ready” [10]. According to Shulman, “we must learn to view aptitude, not as a measure of what can be learned but as a reasonably good predictor of the amount of time necessary for learning” [1]. As a result, individuals may demonstrate an array of performance levels for the various professional activities or competencies expected of them at any given time [11]. A complicating factor is the lack of continuity across the education, training, and the *unsupervised* practice continuum, which provides little opportunity or incentive to revisit and reinforce what has been taught, potentially ameliorating some of the variation in pace of learning.

Complicating the discontinuity, and our refusal to acknowledge that the pace of learning varies across individuals, clinical competency committees often err on the side of transitioning learners to the next phase with weak, no, and even contrary evidence that they are ready [6]. High financial stakes that include education debt for learners, cost of training and remediation for programs, the need to preserve reputations of trainees and programs, and the legal threat of an appeal process contribute to this “failure to fail” problem [7]. The reality, however, of practicing without supervision as trainees transition to the practice years should give us pause.

Logistics of introducing time variability are often touted as one of the greatest barriers to achieving this goal. No doubt the bureaucracy is also a hurdle to be reckoned with, as one considers time dependent licensing, accreditation, and certification. Our focus here, however, will be on the education and training aspects as the first step in getting to a plan for time variable competency-based medical education. We see the logistics reflecting more deeply rooted problems, including lack of: 1) agreement on specific markers of achievement for transitioning from one phase to the next, 2) education handovers that provide a meaningful picture of both learners’ strengths and improvement opportunities, and 3) compassionate off ramps for those needing an alternative career [12,13]. Based on one author‘s (CC) experience, there are many reasons for career choice changes, including but not limited to, misalignment between family expectations and individual choices, increasing debt burden, and inability to meet performance expectations. Alternate careers should align with the reason for the change and may include desired fields outside of medicine, as well as other healthcare careers that are less of a finacial drain or better align skills of the individual with the competencies expected of the professional.

Based on the myriad challenges of implementing time variable, fixed outcome competency-based medical education, we propose using a lens of complexity science to better understand and manage expectations as a starting point. Zimmerman et al describe the differences among simple (e.g., following a recipe), complicated (e.g., sending a rocket to the moon), and complex (e.g., raising a child) phenomenon [14]. To illustrate complexity, we provide an example related to our work: teaching a class. Teachers may use a standard curriculum and methods, give the same assignments and tests, and share grading rubrics. However, the outcome for students is not inherently predictable because they possess different knowledge and skills when the course begins, learn at different rates, vary in their investment to learn, and have different test-taking skills. As this example illustrates, if the goal is uniform outcomes, then the complex intervention to ensure all students reach those outcomes must be predicated on time variability; individualized attention, feedback, coaching; and a program of assessment that facilitates following developmental progress.

We believe that complexity also applies to the process of implementing competency-based medical education based on student variables as well as program variables such as context, culture, priorities, resources, and faculty investment. However, Van Melle et al provide insight into how to begin implementing this complex intervention in their paper entitled “A core components framwork for evaluating implementation of competency-based medical education programs” [15]. By identifying the critical components, they provide the foundation for evaluating all competency-based medical education implementation efforts. With complexity considerations in mind, we lay out a vision, not a recipe, for moving forward with time variable, fixed outcome competency-based medical education, knowing full well that this starting point will and must evolve.

## Envisioning what could be: Conceptualizing readiness in time variable, fixed outcome medical education

Adopting time-variable “readiness” to advance as the basis for transitioning learners across the education, training, and practice continuum has two prerequisites. First, education, training, and practice must move from being merely sequential to functioning as a true continuum. This requires backward visioning from population health needs to postgraduate medical education that ensures trainees are ready to meet those needs by graduation [8]. It also requires that undergraduate medical education be designed to ensure students are prepared to perform the basic building blocks of the postgraduate competencies, setting up each subsequent phase of training and practice to build upon the knowledge and skills of the previous one [16]. The second prerequisite for adopting time-variable training is ensuring that continuous professional development be modeled on “deliberate practice,” in which a growth mindset with intentional learning, coaching, feedback, and repetition become a career long expectation [17,18]. Unlike traditional continuous professional development, where learning activities are self-selected and often not aligned with filling gaps [19], deliberate practice affords one the ability to close gaps, acquire new skills, and set forth on an intentional journey toward expertise [17,18]. With deliberate practice as the goal, backward visioning across the continuum as the path, and armed with a growth mindset, physicians will have the capability to address the evolving population health needs within the context of their practice as they work towards achievement and maintenance of expertise.

We explore each of these prerequisites in detail in the next two sections of the paper.

## Establishing a True Continuum: Replacing *Ready or Not* Decisions with *Ready for What* Decisions

### Logistics: Placing Focus on Ready for What?

In our vision for the future, real time reviews of trainees’ progress would require the development of a readiness scale to replace the annual ready or not decisions used for promotion and transition. For example, a pediatric resident may be ready for indirect supervision when caring for a well newborn, but still require direct supervision when caring for children with behavioral and mental health disorders. Context, as well as complexity of knowledge and skills, impacts performance [20]. Progress is not defined only by readiness for transition to a next phase but readiness for moving from more to less supervision, such as direct (preceptor watching) to indirect supervision (a preceptor available on site or by phone). While the term “unsupervised practice” is often used to describe readiness to transition from training to practice, a more accurate description would be “readiness to practice without supervision,” which encompasses collegial conversations. A readiness scale, based on level of supervision needed, would also help to identify learners’ aptitudes, rates of progression within areas of learning, for the purposes of coaching and remediation [1]. However, no resource comes without limits. If coaching and remediation do not lead to improvement, it is most fair and helpful for learners to understand this as soon as possible. If needed, the use of compassionate off-ramps early in education and training mitigates some of the financial and emotional burdens that learners suffer as a result of having to reimagine a new career path [13]. Predictive analytics, used with caution, care, and human oversight, can be of value in supporting these decisions [21].

### Schedules

An important implication of a time variable system would be a stream of learners who move along an educational continuum at their own pace. Recognizing that some predictability in logistics is almost certainly required for programs just beginning the journey towards time variability, perhaps actual “advancement days” could occur at quarterly intervals. On these days, those trainees who demonstrated readiness for less supervision during the previous quarter could be entrusted with greater responsibilities and those who demonstrate readiness to transition from one phase of the continuum to the next could prepare to do so. Achieving flexibility in scheduling during time-variable pilots has been demonstrated in multiple settings. The Netherlands has been able to develop great flexibility within their training programs. Hoff et al describe the fluidity of their approach to residency training using individual curricula, full and part time training, and an emphasis on team work to provide patient continuity [22]. Overall, the implementation of time-variability, along with entrustable professional activities as the key assessment strategy undergirding their efforts, led to an overall decrease in 3 months of training for most residents [22]. However, these interventions require “a significant change in culture,” as well as ongoing monitoring and adaptation [23]. In Canada, successful implementation of time variable orthopedics residency training in Toronto set the stage for a time variable component to a national initiative “Competence by Design” across all specialties in the country [24,25].

With a continued eye toward feasibility, the ideal for the undergraduate medical education to postgraduate medical education transition would be to have this occur in the same institution for the foreseeable future, with exceptions limited to personal circumstances or lack of a desired specialty at the home institution. Learners remaining at a given institution for the totality of their undergraduate medical education and postgraduate medical education years places greater responsibility on the institution to create effective remediation programs and compassionate off ramps [13]. Enhanced investment in learners may prompt their reciprocal investment in their learning environments and communities, providing an antidote to the burnout that has become so prevalent in medical education and practice [26]. Remaining at the same institution, may also address the perceived need for a single annual transition time to enable and support a group identity. Finally, it would contribute to leveling the playing field for medical students who don’t have the monetary resources to visit residency programs that are a “reach,” needing to focus on target programs likely to accept them.

One author’s experience (CC) with The Association of American Medical Colleges “Education in Pediatrics Across the Continuum” Project supports the concept of undergraduate medical education and postgraduate medical education continuity in the same institution. The project was a 10-year pilot in which learners transitioned from undergraduate medical education to postgraduate medical education and postgraduate medical education to practice based on competence, not time [27]. Medical students being considered for the program expressed interest in staying at their institution for postgraduate medical education whether or not they were accepted to the pilot program [28]. Observations over a decade showed that site continuity: 1) alleviated the competition of the “Match,” allowing trainees to focus on learning as opposed to vying for residency positions, 2) facilitated “responsible” educational handovers” [29,30] ensuring both strengths and areas for improvement were included in the individual learning plan going forward, and 3) obviated the need to audition for and visit other programs, eliminating prohibitive costs and lost educational time associated with these activities.

### Learners

With learning as the goal and time as a resource, learners’ focus could shift from a performance to a growth mindset where learning trumps performance [31]. Real time progress reviews that include constructive feedback, and goal resetting, can be coupled with decisions and plans about increasing responsibilities and transitions. The alternative to a growth mindset, a fixed mindset, is characterized by the belief that ability cannot be changed which negatively impacts attempts to learn. It has been identified as a factor in increasing shame which subsequently reinforces a fixed mindset [32,33]. A growth mindset also allows learners to constructively and continuously measure against newer versions of themselves rather than destructively measuring themselves against peers. The observations of one author (CC) during the Education in Pediatrics Across the Continuum Pilot showed that lessening competition among learners enabled them to request and offer help more freely, thus enriching experiences and enhancing satisfaction in their learning and work. Klasen et al have also noted that allowing trainees to make rectifiable mistakes, followed by debriefing and follow-up conversations about lessons learned, can contribute to growth and learning [34]. The conversations were key, again reinforcing the need for connection to colleagues as fundamental to our well-being and serving as the seed from which resilience grows [26].

## Education and training as preparation for *deliberate* rather than *unsupervised* practice

Unsupervised practice traditionally refers to a credentialed physician who does not require oversight from another physician. The reality is that we often practice with support from colleagues and information resources because we do not and cannot know everything. Many find it more rewarding, and even vital to their practice, to share interesting and complex cases with colleagues. Deliberate practice builds on this foundation through one’s commitment to a growth mindset, and the drive to continuously improve by seeking and incorporating feedback into practice throughout one’s career. It is the only pathway to achieving and maintaining expertise [35].

### Deliberate practice holds the key to a time variable continuum

Currently, learning during the practice years following training can either be characterized as a self-regulated process or in some countries, such as Canada and the United States, a nationally regulated process, with opportunity for some individual choice. The fatal flaw within these processes is that we are all poor at self-assessment, resulting in gaps that widen rather than close [36]. Nationally regulated processes require that physicians select from a menu of learning activities, not necessarily specific to one’s practice or gaps, and complete the required number within a specific timeframe. The United States also requires online knowledge testing as part of maintaining certification. In contrast, *deliberate* practice, addresses one’s specific gaps and new learning needs via continuous cycles of coaching, feedback, and repetition that leads to improving the quality of care within the context of one’s practice.

The literature documents the graduation of trainees who have clear gaps in skill sets [37,38,39]. A recent study suggests that for some professional activities, developed by the community for their own subspecialty, program directors would graduate fellows who still require direct or indirect supervision to perform them, particularly in the procedural subspecialties where experience must be gained by deliberate practice [40]. This acknowledgement is critical to dispelling the myth that physicians are truly prepared for unsupervised practice once training is complete. It also reinforces the critical need to ensure that entrustment decisions are based on both retrospective (e.g., data available about past performance) and prospective (e.g., informed inference about readiness to practice in future not fully knowable contexts) components. The key components of the latter being help-seeking and other elements of trustworthiness [6,41].

### The implications of deliberate practice on medical education, training, and continuous professional development

As noted above, a growth mindset during medical education and training is critical to a career of deliberate practice, which is predicated on continual growth on the never-ending journey towards achievement and maintenance of expertise. Backward visioning leads us to also consider the impact that deliberate practice may have on undergraduate medical education and postgraduate medical education. The longevity of the deliberate practice years, as well as the need for new knowledge and skills as one’s patients and practice evolve, provides the opportunity to rethink the utility and consequences of ready or not summative decisions at the completion of training [42]. In fact, van der Vleuten et al advocate that any assessment effort be both formative and summative, only to varying degrees and instead propose the terms “high and low stakes” [43]. We suggest taking this a step further by using the terms *higher* and *lower* stakes decisions, which better aligns with the concept of a spectrum of decisions characteristic of a readiness scale. To bring to life the time variable, fixed outcome design that competency-based medical education is predicated upon, we envision the possibility of turning a single summative ready or not decision into a series of *lower* stakes progress reviews punctuated by *higher* stakes decisions that predict readiness for increasing responsibility. Each review would provide feedback and direction to assist with attaining the next step. Points of transition across the education, training, and practice continuum would require higher stakes decisions. Transition to practice would be based on both retrospective assessment data (direct observations over time) and a prospective entrustment decision based on an individual’s ability to extrapolate from familiar to unfamiliar care situations and seek help when needed [6].

Anders Ericsson [35], who described the road to deliberate practice, makes clear that the journey requires great focus, relentless repetition of tasks that incorporate feedback on performance, and undying determination. Importantly, experience alone, without reflection, feedback, and relentless practice, leads to efficiency but not expertise [44]. Deliberate practice provides the potential to replace current methods of continuous professional development with a meaningful road to expertise for individual practitioners and the continual improvement of care quality for the unique and evolving patient populations they will serve.

Expecting physicians to engage in deliberate practice means educating with this in mind. Reflection, coaching, feedback literacy [45], and assessment are critical skills not only for faculty but for all learners, who will be called upon to engage in these activities with peers throughout their careers.

## Travelling the Continuum

If we heed the considerations we have laid out, we simply must blur the single pre-ordained lines of transition across the continuum of education, training, and practice with flexibility afforded by ranges of time. One of the strongest recommendations from the scoping review by Yardley et al is that “transitions should not be viewed as one moment in time: career trajectories are a continuum” [46]. The authors also “encourage progressive independence by offerining a sliding scale of decreasing supervision alongside demonstrating increasing trust” [46]. When learners meet all pre-established standards of a given phase, they are ready for transition to the next phase. In the Education in Pediatrics Across the Continuum program, most learners transitioned to postgraduate medical education during the first or mid-second semester of the fourth and final year of medical school and completed residency training three to six months earlier than the thirty-six required months [27]. Likewise, variable transition dates, either earlier or within the typical time frame, were the hallmark of a successful pilot program in orthopedic surgery at the University of Toronto [24]. In addition to meeting pre-establsihed standards, transition to practice should be based on a judgment of the learner’s capacity to apply knowledge and skills in unfamiliar situations and the trust to invoke help when needed [6].

## Kairos: The time is now

“Chronos” was the Greek god of time, representing empirical time as we know it: the past, present, and future. However, the Greeks also marked time by “Kairos,” that is opportunistic time defined as a key moment when required action must be taken to yield desired results [47]. Implementing time variable education and training that affords all learners the ability to achieve the requisite knowledge and skills to improve population health is long overdue. Ready or not, *now* is the “Kairos” for the medical education community to lead this effort.
